# Patient-reported experiences of cognitive difficulties and their impact on daily life in narcolepsy type 1

**DOI:** 10.3389/fneur.2026.1760952

**Published:** 2026-03-04

**Authors:** Kiran Maski, Kathryn M. Pfeiffer, Meryl Brod, Robert D. Latzman, Sarah L. Bermingham, Paul Maruff, Brian T. Harel

**Affiliations:** 1 Boston Children’s Hospital Harvard Medical School, Boston, MA, United States; 2 The Brod Group, Mill Valley, CA, United States; 3 Takeda Development Center Americas, Inc., Cambridge, MA, United States; 4 Takeda Pharmaceuticals U.S.A., Inc., Cambridge, MA, United States; 5 Cogstate Ltd., Melbourne, VIC, Australia

**Keywords:** cognitive difficulties, cognitive symptoms, qualitative interviews, narcolepsy, NT1

## Abstract

**Background:**

The cognitive difficulties experienced by individuals with narcolepsy type 1 (NT1), a rare and chronic neurological disorder, are understudied, with limited knowledge of their consequences in daily life. Here, we investigated the nature of cognitive difficulties and their consequences for daily life from the perspective of adults with NT1.

**Methods:**

In-depth, qualitative interviews were conducted with adults diagnosed with NT1 residing in the United States. Participants were recruited through (1) a patient advocacy organization (via social media and website); (2) a professional market research firm; and (3) participant referrals. Individual interviews were conducted by telephone, following a semi-structured guide, and lasted approximately 90 min. Qualitative analysis used an adapted grounded theory approach to identify key conceptual themes related to cognitive difficulties and their impacts on daily life.

**Results:**

Of 46 participants, most reported experiencing some cognitive difficulties, with the most common being trouble remembering and difficulty with focus or sustained attention. Most participants characterized their difficulties with cognition as moderate or severe and reported these occurring daily. The qualitative findings informed the development of a conceptual model depicting cognitive difficulties and their broad impact on functioning and well-being in adults with NT1.

**Conclusion:**

Cognitive difficulties in adults with NT1 are frequent, severe, and described as interfering with daily life activities and well-being. These data highlight a clear need to assess cognitive function in people with NT1 and identify treatments that address NT1-associated cognitive symptoms.

## 1 Introduction

Narcolepsy type 1 (NT1) is a chronic and rare neurological disease characterized by a pentad of symptoms that include excessive daytime sleepiness (EDS), cataplexy, disrupted nighttime sleep, hypnagogic and hypnopompic hallucinations, and sleep paralysis (1, 2). NT1 is caused by a substantial and selective loss of orexin-producing neurons in the lateral hypothalamus (3). Orexins play a key role in sleep–wake regulation and maintenance of wakefulness through stimulation of brain regions that maintain alertness; thus, their loss can result in significant disruption in sleep–wake states. Orexin loss is further associated with alterations in automatic behaviors, mood and emotional regulation, reward processing, and cognition (4).

Cognitive symptoms are increasingly recognized as important and disruptive in NT1 (2). Cognitive symptoms associated with NT1 can have substantial negative impacts on many aspects of patients’ lives, affecting daily function, educational attainment, work performance, and relationships, as well as emotional well-being and social interactions (5–8). Across surveys of adults with narcolepsy, cognitive symptoms are consistently rated as among the most burdensome of the disease (5, 9). In one study, 36% of people with NT1 reported that cognitive symptoms were the symptom they most wanted a medication to improve (9).

Both objective and subjective data contribute to understanding cognitive symptoms in NT1. Neuropsychological tests are objective, performance-based assessments of cognition that allow characterization of the nature and extent of cognitive impairment in NT1. The assessment of individuals’ subjective understanding of their cognition, as well as the impact of perceived difficulties in cognition on their day-to-day function provides a strong basis for understanding how individuals with NT1 experience cognitive symptoms in their daily lives. It also allows for insights into how cognitive symptoms contribute to NT1-related disability or disease severity (10–12). Throughout this article, “cognitive difficulties” is used to reflect how adults with NT1 describe their cognitive symptoms; whereas “cognitive impairment” refers to findings from objective neuropsychological assessments of cognition.

Despite growing recognition that many individuals with NT1 report experiencing cognitive difficulties, and that these have a major impact on their daily lives, there is limited understanding of these experiences. Consequently, there is a lack of conceptual frameworks to organize individuals’ reports of cognitive difficulties and their impact on daily life. There is a need to better understand the nature and frequency of cognitive difficulties experienced by people with NT1, including how these impact daily function and quality of life in the real world. This qualitative study aimed to address this gap by exploring how adults with NT1 describe the nature and frequency of their cognitive difficulties and how these difficulties impact their daily activities, work, education, and emotional and social well-being.

## 2 Methods

### 2.1 Study design and participants

This was a secondary analysis of a broader qualitative study of adults diagnosed with NT1 (publication in development). The present analysis focused specifically on participants’ descriptions of cognitive difficulties and their influence on daily life and well-being, including impacts on work, education, emotions, relationships, and identity. The study sample included 46 adults aged 18 years or older residing in the United States with self-reported EDS and cataplexy, and documented proof of NT1 diagnosis from a medical provider or NT1-specific prescription medication, or self-reported NT1 diagnosis informed by a physician.

Participants completed individual, 90-min, semi-structured interviews, between August and December 2022, designed to elicit detailed descriptions of the full range of NT1 symptoms and their impacts. The interview discussion guide was informed by an unpublished scoping study of patient-created content, which explored NT1 symptoms and their impacts using publicly available content (including memoirs, podcasts, blogs, videos, and forum discussions) and generally included open-ended questions and follow-up probes. This was followed by a virtual focus group conducted in May 2023 with several of the original interview participants, to provide feedback on interview findings and provide input into development of conceptual models of symptoms and impacts associated with NT1. Focus group participants were asked to comment on the relevance and completeness of the conceptual model, identify any elements they felt were missing, and assess the accuracy and appropriateness of the language used.

The study received institutional review board approval from the WCG Institutional Review Board (Puyallup, Washington; Study Number 1331629). Informed consent was obtained from all participants. Additionally, a researcher living with NT1 (SLB) was involved in all stages of the research process, including study design, data interpretation, and writing. This researcher did not conduct interviews, the focus group, or thematic coding to maintain reflexivity and reduce potential bias.

### 2.2 Data collection

Demographic information was collected prior to the interview via an emailed demographic form. Interviews were conducted by 1 of 3 experienced qualitative interviewers using a semi-structured discussion guide informed by an unpublished scoping study of patient-created content, and the focus group was conducted virtually using a professional research platform and lasted 93 min.

Interviews specifically asked about cognitive symptoms, and participants were probed further when cognitive difficulties were mentioned spontaneously. Participants were asked “What signs or symptoms have you experienced due to narcolepsy?” with a probe for cognitive symptoms (eg, issues with remembering things, focus/concentration, thinking clearly). If not mentioned spontaneously, all participants were probed on whether they experienced any difficulty with thinking clearly, concentrating/focusing, remembering things, forming thoughts, maintaining their attention, learning new things, or other cognitive issues. When “brain fog” was spontaneously mentioned, interviewers were instructed to probe for meaning of the term (eg, What does “brain fog” mean to you? Can you describe that symptom for me?). Additional questions asked about the frequency and severity of the reported difficulties and how they affect daily life or functioning, including education, work, and well-being. As the interviews were semi-structured, interviewers followed the lead of the participant, and not all questions were asked in the exact same way or in the same order. All interviews were audio recorded, transcribed verbatim by a professional translation firm, and anonymized.

### 2.3 Data analysis

Analysis of interview transcripts was informed by an adapted grounded theory approach, an established qualitative research methodology in which theoretical models are developed based on the analysis of data (13, 14). Transcripts were analyzed and coded by an experienced qualitative researcher (KMP) using a coding scheme developed initially based on the interview guide. The transcript coder conferred with the research team for input on the coding scheme, revisions and conceptual model development, and to ensure consistent coding and accurate capture of participant experiences.

Coding followed an iterative process that allowed emerging concepts to be incorporated into the coding scheme. Codes were continuously refined and organized into broader themes and subthemes, and a coding log documented all additions and revisions. When a new code was introduced, previously coded transcripts were re-examined for the new theme to ensure consistency in coding; thus, excerpts related to cognitive symptoms or related impacts were reviewed multiple times. Any cognitive difficulties that a participant described to be part of their conception of brain fog were co-coded with mentions of brain fog. When participants spoke of their cognitive difficulties using general terms, impacts were coded for the cognitive difficulties the participant reported to ensure that all impacts associated with those cognitive difficulties were captured. Thematic saturation, the point at which important themes and insights are no longer emerging from the data, was assessed in the chronological order of interviews using a saturation grid. Saturation was considered reached once 95% of concepts had been covered in the interviews.

A conceptual model focused on the experiences of cognitive difficulties and their consequences in people with NT1 was developed collaboratively by the research team and refined further based on feedback from focus group participants.

Qualitative coding and data analysis were conducted using dedoose (15), a qualitative and mixed methods research web application. Descriptive statistics were used to analyze demographic and health characteristic data; frequencies and percentages were reported for nominal/ordinal variables and means/medians with standard deviations, ranges, and interquartile ranges were reported for continuous variables. Reporting adhered to the O’Brien et al. standards for reporting qualitative research (16) and followed the Consolidated Criteria for Reporting Qualitative Research (COREQ) 32-item checklist (17).

## 3 Results

### 3.1 Participant characteristics

Forty-six participants [mean (SD) age 34.7 (11.3) years, 72% female] completed individual interviews (Table 1), 7 of whom also took part in the focus group. Participant-reported racial/ethnic background (not mutually exclusive) included White/Caucasian (83%, *n* = 38), Black/African American (17%, *n* = 8), American Indian or Native Alaskan (7%, *n* = 3), Asian (7%, *n* = 3), and Hispanic/Chicano/Latino (7%, *n* = 3). All US geographic regions were represented in the sample. Most participants reported good to excellent overall health status (67%, *n* = 31), and most used prescription medication(s) to manage NT1 symptoms in the previous month (89%, *n* = 41). Detailed participant background characteristics are provided in Table 1.

**Table 1:** Participant demographic and background characteristics.

Characteristic	Participants (*n* = 46)
Age, years	
Mean (SD)	34.7 (11.3)
Median (range)	34.0 (18–67)
Age at NT1 diagnosis, years	
Mean (SD)	26.8 (11.2)
Median (range)	25.0 (7–65)
Sex	
Female, *n* (%)	33 (72)
Racial/ethnic background, *n* (%)^a^	
White/Caucasian	38 (83)
Black/African American	8 (17)
American Indian or Native Alaskan	3 (7)
Asian	3 (7)
Hispanic/Chicano/Latino	3 (7)
Other not listed	1 (2)
Highest level of education completed, *n* (%)	
Less than high school	1 (2)
High school or equivalent	2 (4)
Vocational or technical degree	2 (4)
Some college	12 (26)
College or university degree	18 (39)
Graduate or professional degree	11 (24)
Current work status, *n* (%)	
Work full-time for pay	16 (35)
Work part-time for pay	8 (17)
Student and work part-time for pay	5 (11)
Student (not working)	5 (11)
Retired	1 (2)
Not working	11 (24)
Not working or working less because of narcolepsy, *n* (%)	28 (61)
Medication	
Used prescription medication to manage narcolepsy in past month, *n* (%)	41 (89)

Percentages may not add to 100 due to rounding. ^a^Responses are not mutually exclusive. SD, standard deviation.

The focus group consisted of 7 adults (5 women and 2 men), aged from 22 to 47 years, with diverse backgrounds. Five participants identified as white, 1 as Asian, and 1 reported multiple racial/ethnic backgrounds, including American Indian/Native Alaskan, Black/African American, and White. Three participants reported a college or post-graduate degree, and 4 had education below college degree level. In terms of work status, 4 worked full-time, 1 worked part-time and was a student, and 3 did not work for pay.

### 3.2 Cognitive difficulties in NT1

#### 3.2.1 General experiences

All but one of the 46 participants interviewed reported that they experienced cognitive difficulties associated with NT1. Most noted the onset of cognitive difficulties in adolescence or adulthood, though a quarter reported childhood onset (age <10 years). Of those participants reporting on symptom frequency and severity, nearly three-quarters reported experiencing cognitive difficulties daily and nearly half rated them as severe.

**Figure 1:**
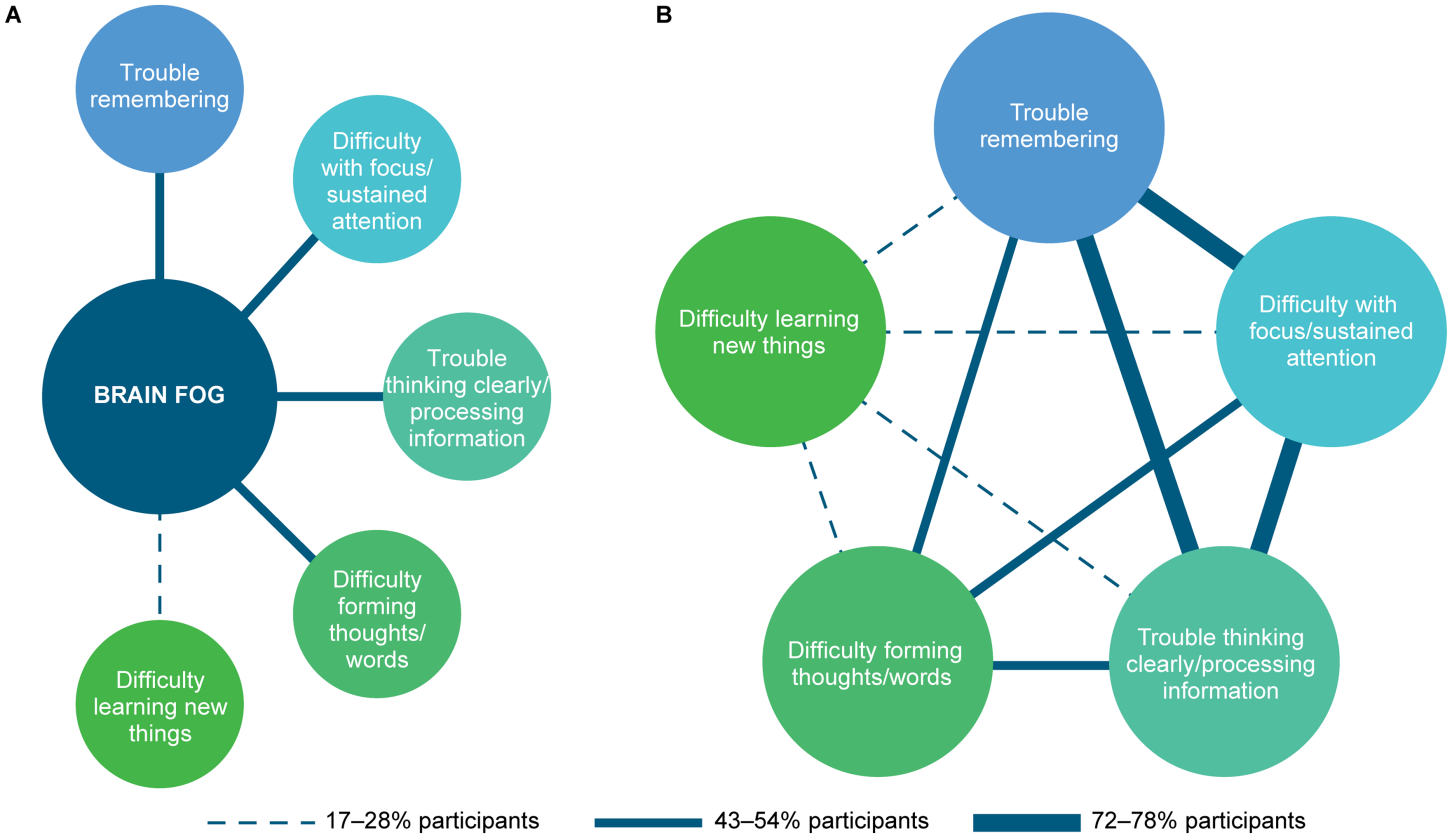
Co-occurrence of reported cognitive difficulties associated with narcolepsy type 1. **(A)** Cognitive difficulties for which the descriptive term “brain fog” was used. **(B)** Inter-relationships between cognitive difficulties reported. Lines indicate the percentage range of participants who reported both difficulties.

Based on the analysis of participant interview data, descriptions of cognitive difficulties were coded into 5 key themes: (1) trouble remembering; (2) difficulty with focus/sustained attention; (3) trouble thinking clearly/processing information; (4) difficulty forming thoughts/words; and (5) difficulty learning new things. The descriptive term “brain fog” was also used by some participants; when probed to describe what brain fog meant to them, participants always referred to other cognitive difficulties included within the 5 key themes. They often endorsed 2 or more of these cognitive difficulties to define their experience of brain fog. Relationships between the term “brain fog” and the specific cognitive difficulties reported by participants to describe this term are summarized in Figure 1A. Although each cognitive difficulty, including those associated with brain fog, is discussed separately, co-occurrence was frequent (Figure 1B), with most participants having experienced 3 or more of the reported cognitive difficulties. Participant experiences of each cognitive difficulty and brain fog are described in detail below, and additional selected quotations are provided in Supplementary Table 1.

#### 3.2.2 Trouble remembering

**Figure 2:**
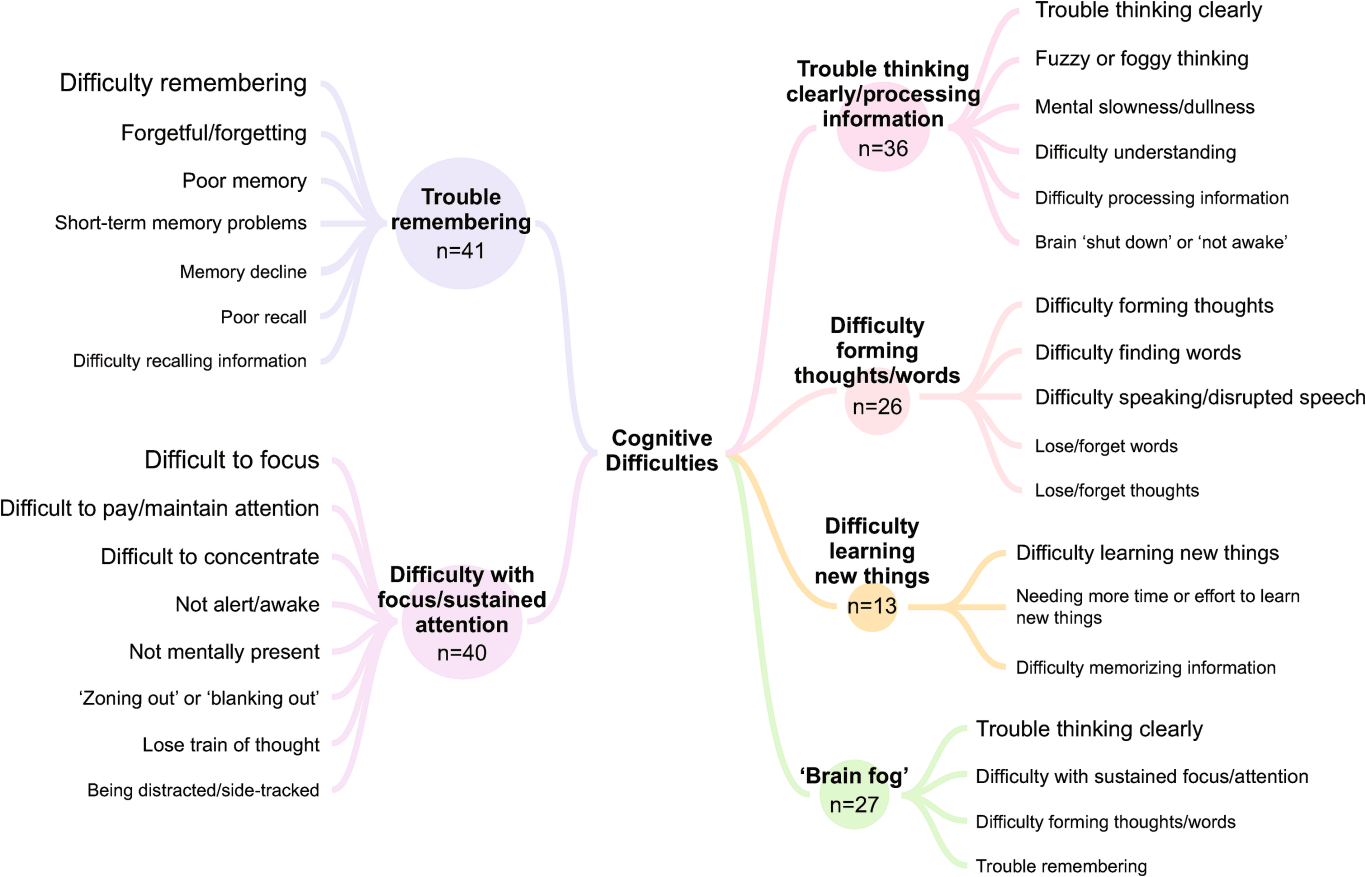
Reported cognitive difficulties and brain fog associated with narcolepsy type 1. The term “brain fog” was a more general concept that participants often used to describe various cognitive difficulties, most frequently trouble thinking clearly/processing information and difficulty with focus/sustained attention.

The most frequently reported theme associated with NT1 was trouble remembering or memory problems. Most participants with this complaint described general difficulty or trouble remembering things, such as names, dates, appointments, conversations, and daily tasks. Many participants also discussed being forgetful or forgetting things, having poor memory and short-term memory problems, while some described memory decline or worsening memory over time, having poor recall, and difficulty with retaining information (Figure 2).

*“I have just a hard time…remembering things. If I'm tired at the moment and someone comes in and asks me to go to the store and buy like three different things…sometimes, depending how sleepy I am, I probably won't even remember them asking me to do something.”* (female, age 29 years).

*“… I'll forget. I can be in the middle of something and forget what I was doing…I'll think, you know, "Hey, I need to do this later," but by the time later runs around I may forget.”* (female, age 32 years).

Memory loss or long-term memory problems were reported more rarely. In addition to medication, participants discussed using a variety of coping strategies to help manage their memory problems, most commonly calendars/reminders or notes/lists, while some relied on help from family and friends.

#### 3.2.3 Difficulty with focus/sustained attention

Difficulty with focus or sustained attention was reported by most participants. Beyond frequently described trouble with focus, paying attention, or maintaining attention, other descriptions raised commonly included difficulty concentrating or being unable to concentrate for long periods of time, being less alert/awake, and not being mentally “present.” Some participants also discussed “zoning out” or “blanking out,” losing their train of thought or difficulty grasping/holding on to thoughts, and being distracted or sidetracked (Figure 2).

*“…when it comes to being able to pay attention, you know, I'll often find that…I'll have to ask my kids or my partner to repeat themselves.”* (male, age 41 years).

*“…I just can't…concentrate for very long. My mind wanders constantly.”* (female, age 56 years).

The coping strategies mentioned most frequently for managing difficulty with focus/sustained attention were movement or stimulating activity, daytime naps, and use of calendars, reminders, notes, or lists.

#### 3.2.4 Trouble thinking clearly/processing information

More than three-quarters of participants discussed difficulty with thinking clearly or processing information. They most frequently reported having trouble thinking clearly or “straight,” general difficulty thinking, being unable to think, or having thoughts that were mixed up or jumbled/out of order. Participants also often described experiencing fuzzy, foggy, hazy, or cloudy thinking, a mental dullness/slowness or not being mentally sharp, difficulty understanding/comprehending, difficulty processing, absorbing, or registering information, and feeling that their brain was “shut down,” not awake, or not working (Figure 2).

*“…if I'm about to fall asleep, I'm not thinking clearly…if I even have like a really disrupted night's sleep, I feel like my thoughts are really more run by emotion than anything else because I'm just so tired like just trying to get through the day…”* (female, age 30 years).

*“I just feel slow. My… brain doesn't register things as quickly. I don't process as quickly. I have to repeat things often. I forget things often…”* (female, age 28 years).

Difficulties mentioned less frequently included mental confusion and trouble with complex thinking or reasoning. The most commonly reported strategies for managing or coping with difficulties with thinking clearly or processing information were daytime naps, use of calendars, reminders, notes, or lists, and daily scheduling or planning.

#### 3.2.5 Difficulty forming thoughts/words

More than half of participants discussed having difficulty forming thoughts or words. Of these, participants most often described experiencing difficulty with forming thoughts, finding words, and speaking or having disrupted speech (Figure 2). Participants also mentioned losing/forgetting words and losing/forgetting thoughts.

*“…I sometimes feel like I have a hard ti-- like I was saying earlier, I have a hard time forming thoughts…”* (female, age 20 years).

*“…I'm consistently, uh, mumbling my words, or not being able to get out sentences as coherently as I'd like to.”* (male, age 35 years).

The most often noted coping strategies participants used to manage difficulty forming thoughts/words included use of calendars, reminders, notes, or lists and social support or help.

#### 3.2.6 Difficulty learning new things

Several participants reported difficulty learning new things. These participants most often described having general difficulty learning new things, needing more time or effort to learn new things, and difficulty memorizing information or retaining what they learned (Figure 2).

*“I think that I am still able to learn new things. I think it just sometimes takes longer or, um, I need to revisit it…more…[frequently than] someone else.”* (female, age 27 years).

*“…in school I did well if I un-understood things…if I can understand it I got it, but I can't memorize things. Memorizing does not work for me. I can't remember anything.”* (female, age 39 years).

Participants often attributed their difficulty in learning new things to other cognitive difficulties, including trouble remembering, difficulty with focus/sustained attention, and trouble thinking clearly/processing information. Two participants mentioned the use of caffeine, energy drinks, or nicotine for managing difficulty learning new things.

#### 3.2.7 “Brain fog”

More than half of participants reported experiencing “brain fog” associated with NT1 (Figure 2). When asked what they meant by “brain fog,” participants responded with terms such as “cloudiness,” “spaciness,” “being groggy,” and “hazy.”

*“…it's just foggy head. Having trouble…thinking or latching onto what I'm trying to concentrate on, or articulate, or whatever. So just again, you know, manifesting as losing time when I'm trying to concentrate or focus on something that requires a lot of attention.”* (female, age 47 years).

*“…kind of hazy feeling. Like…I can't focus, can't grab a thought, can't stay focused on anything. Or I'll lose words…”* (female, age 56 years).

In response to probing participants who mentioned “brain fog” to explain the term further, all discussed one or more of the 5 key cognitive difficulties identified in their accounts of “brain fog”; most participants described 2 or more cognitive difficulties, while several associated brain fog with only 1 cognitive difficulty. The cognitive difficulties most frequently associated with brain fog were trouble thinking clearly/processing information and difficulty with focus/sustained attention (Figure 2). Participants reported several coping strategies for managing brain fog, most commonly using calendars, reminders, or notes/lists, taking daytime naps, and daily scheduling or planning.

### 3.3 Impacts associated with cognitive difficulties

**Figure 3:**
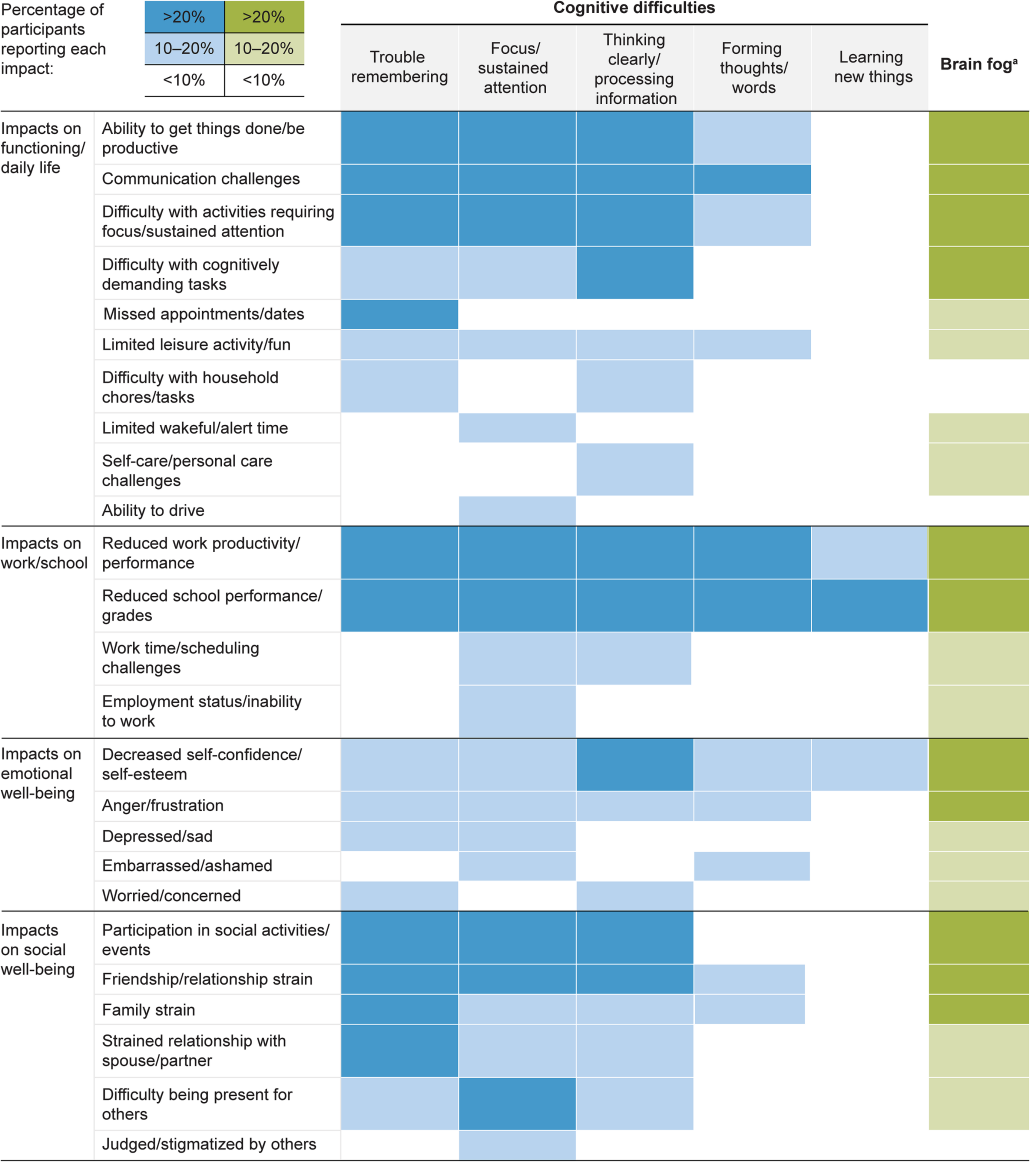
Most frequently reported impacts of cognitive difficulties and brain fog in narcolepsy type 1. Dark blue/green shading indicates impact reported by ≥20% of participants who experienced the cognitive difficulty. Light blue/green shading indicates impact reported by 10 to <20% of participants who experienced cognitive difficulty. No shading indicates impact reported by <10% of participants who experienced cognitive difficulty. ^a^Participants used the term “brain fog” to refer to various cognitive difficulties.

Participants described a wide range of impacts they attributed to cognitive difficulties, including those associated with their experience of “brain fog.” These impacts were categorized into 4 domains: (1) functioning and daily life, (2) work and school, (3) emotional well-being, and (4) social well-being and relationships. Frequently described impacts associated with each cognitive difficulty are presented by impact domain in Figure 3, and additional selected quotations are provided in Supplementary Table 2.

#### 3.3.1 Impacts on functioning and daily life

Approximately three-quarters of participants reported that cognitive difficulties associated with NT1 affected their daily functioning. The most frequently mentioned impact on daily life was communication challenges, such as difficulty having or remembering conversations.

Communication challenges were frequently discussed in relation to difficulties with memory, focus/sustained attention, thinking clearly/processing information, and forming thoughts/words.

*“…when that daytime sleepiness hits…sometimes I will kind of stop mid-sentence if I'm talking to somebody or explaining something because I forget what I'm going to say.”* (female, age 35 years).

*“I would say [I experience] a combination of difficulty thinking, difficulty just processing…when it's bad …I'll have to ask people that I'm speaking with to, like, slow down just because I can't really pick up everything that they're saying if they're talking at an even moderately rapid pace.”* (male, age 39 years).

Participants also commonly reported interference in their general ability to get things done or be productive and difficulty with activities requiring focus or attention, such as reading, writing, watching television, or listening to podcasts or audio books. These issues were frequently attributed to problems with memory, focus/sustained attention, and thinking clearly/processing information.

*“…the sleepiness makes it so I basically can't do anything. And then, even when I'm not sleepy, I kind of don't want to do anything because I just can't focus on anything or remember what I was supposed to do…”* (male, age 34 years).

*“I can't focus…I'll listen to a podcast, or l-listen to an audiobook, or watch a show…I just can't like concentrate for very long. My mind wanders constantly.”* (female, age 56 years).

Some participants also reported difficulty with cognitively demanding tasks such as planning, decision-making, and complex reasoning, which they often attributed to difficulty thinking clearly or processing information.

*“…my cognitive ability is just like non-existent at this point. It's…tough for me to…think about what I want to do or the next thing that I want to do so I kind of get stuck a lot…”* (female, age 39 years).

Missed appointments or dates were also discussed by several participants, and this was related primarily to trouble remembering.

*“I can be very…forgetful and -- Yeah. Just certain -- certain things if I have to remember or have to go to, then I'll…just kind of space out and forget about it.”* (female, age 29 years).

Less frequently mentioned impacts on daily life and functioning included difficulty with household tasks or chores, limited leisure activity and fun, having limited wakeful/alert time, interference with driving ability, and self-care/personal care challenges.

*“I often will just completely forget about something if I'm -- if I'm tired. Um, going to the grocery store, that type of stuff.”* (female, age 40 years).

*“I'm really big into playing fantasy baseball. And it's kind of ironic because now I have more time. But I'm able to do less…I have a hard time concentrating.”* (male, age 52 years).

#### 3.3.2 Impacts on work and education

Almost two-thirds of participants reported that cognitive difficulties impacted their work or career. Among these, nearly all described reduced work productivity or performance, which was often reported in relation to difficulties with memory, focus/sustained attention, thinking clearly/processing information, and forming thoughts/words.

*“There's a lot of loss of memory…where I put things…not being able to memorize where I put stuff. While I'm working…I'll work on a computer, and I'll forget what I'm doing and push the wrong buttons.* (male, age 43 years).

*“[I have] to re-read things [at work]. Like there are some days when my brain fog is so intense that…I am not understanding anything that I'm reading at all…And my work is -- I have to…abstract very specific things from the items that I read. And if I…am not interpreting them correctly…it's going to affect my efficiency…And my accuracy.”* (female, age 37 years).

Less frequently mentioned work-related impacts included work time/scheduling challenges (eg, difficulty working at certain times of day), employment status (eg, job loss, inability to work), missed workdays or time, and strained workplace relationships. Impacts of cognitive difficulties on schooling or education were also described by two-thirds of participants. Among these, reduced school performance or grades were recalled by nearly all, and was attributed to all 5 cognitive difficulties.

*“…I couldn't do the full class load in college…I tried to split things up but it was hard to get up in the morning to get to class… I'd fall asleep during class. I wasn't processing information well. I wasn't understanding stuff because I was just too tired. And I just felt like I was stupid but it was just that I really couldn't focus.”* (female, age 40 years).

*“…even whenever I was awake, I just couldn't concentrate on what the lesson was. Uh, and even if they got me to pay attention per se, you know, keep watching what the person is doing on the board, uh, it wasn't really sinking in. Like I wasn't able to effectively learn even if I was watching what they were doing.”* (female, age 27 years, not treated).

#### 3.3.3 Impacts on emotional well-being

More than half of participants reported emotional impacts related to the cognitive difficulties of NT1. The specific impacts most frequently described were reduced self-confidence or self-esteem, which were most often associated with trouble thinking clearly/processing information.

*“I feel like a failure actually. So, it's affected my emotional wellbeing… we've been packing stuff to go on a trip, a 5000-mile trip. And…we used to do this constantly…And I'm a packer. I make lists. And this has taken me a month and a half to even think about what to [pack]… I can't function mentally well anymore. Everything's a struggle…”* (female, age 67 years, not treated).

*“…I keep saying, my confidence, and that's a big part of [my daily life]. Like, I don't like to do certain things, socialize with a certain [number] of people…because I'm afraid, like, I might slip into a fog and not know what I'm talking about or something like that in the middle of a conversation…”* (male, age 43 years).

Some participants also described feelings of embarrassment or shame and anger and frustration because of their cognitive difficulties.

*“I feel like I can come off really stupid to someone when I'm bumbling my words and so, I'm like, "Oh, sorry, like my brain's not working today." …that's my way of coping with it because I get really embarrassed.”* (female, age 27 years).

*“So it just feels like my brain is full of fuzz and fog, and like I'm walking around in a foggy field and I can like kind of make things out in front of me but not really. And like I'll reach out to touch something, and I can't grab it. Like that's what brain fog is for me. And it's really frustrating because I feel like, when I am alert, when my meds have kicked in, like I'm a very articulate and engaging person.”* (female, age 37 years).

Less frequently mentioned emotional impacts included feelings of sadness or depression, worry or concern, irritability or annoyance, general upset or hurt, and overwhelm or stress.

*“…it's heartbreaking to not be able to remember things, even special things that people around me can.”* (female, age 46 years).

*“I worry about [memory issues] a lot. I worry that I am not…I run a significant part of a company. And so there's not a lot of time to where I don't have a lot of things I do need to remember. I always need to remember a ton of things…it worries me. I worry that I'm going to drop the ball on things. And very rarely do I. But it's definitely a source of concern. I don't ever want to look incompetent to my boss.”* (female, age 42 years).

#### 3.3.4 Impacts on social well-being and relationships

Almost two-thirds of participants reported that cognitive difficulties associated with NT1 negatively impacted their social well-being and relationships. The most frequently discussed impacts were friendship/general relationship strain and strained relationships with spouses or partners. Friendship strain was commonly linked to difficulties with memory, focus/sustained attention, and thinking clearly/processing information, while partner relationship strain was most often associated with difficulties with memory and thinking clearly/processing information.

*“…I have forgotten important dates. I've forgotten or, you know, just been -- either forgotten or just been too, uh, sort of floating off into space to go to doctor's appointments that I needed to go to. Or, uh, you know, I would miss something that a friend invited me to. And you know, that would strain that relationship.”* (male, age 34 years).

*“I have a hard time…remembering things or focusing. And so…that can definitely impact, like, conversations with other people and holding friendships.”* (female, age 24 years).

Family strain was attributed most frequently to trouble remembering, while difficulty being mentally present for others was often attributed to difficulty with focus/sustained attention.

*“…when it comes to being able to pay attention…I'll often find that…I'll have to ask my kids or my partner to repeat themselves. They understand what I'm going through as best as they can. But I know it's a frustrating moment for them as well if they've just gotten into a…particularly…tough topic, or they're trying to explain something that they're very excited about only to find out that…I kind of blanked out a few minutes ago. So that's kind of the biggest challenge there.”* (male, age 41 years).

Among those who reported social and relational impacts, over half said cognitive difficulties interfered with participation in social activities or events. As with impacts on relationships, this was most often attributed to trouble with memory, difficulty with focus/sustained attention, and trouble thinking clearly/processing information.

*“There are definitely times when I, you know, will say no to social engagements because…I feel like I can't get through them, can't be like present there. There are times where I'll…leave something early because I need to go rest.”* (female, age 20 years).

Some participants reported that they felt judged or stigmatized by others due to their cognitive difficulties.

*“…going to [daughter’s] gymnastics meet…I can handle going there. I just avoid trying to sit with the team moms because I just, all the chatter and everything going on I just get uncomfortable if I start zoning out or if I'm-I'm tired and not contributing to the conversations and then they just sort of think that I'm, I don't know, I feel like they-they think I'm just not being social.”* (female, age 40 years).

### 3.4 Development of a theoretical model of the impacts of cognitive difficulties

**Figure 4:**
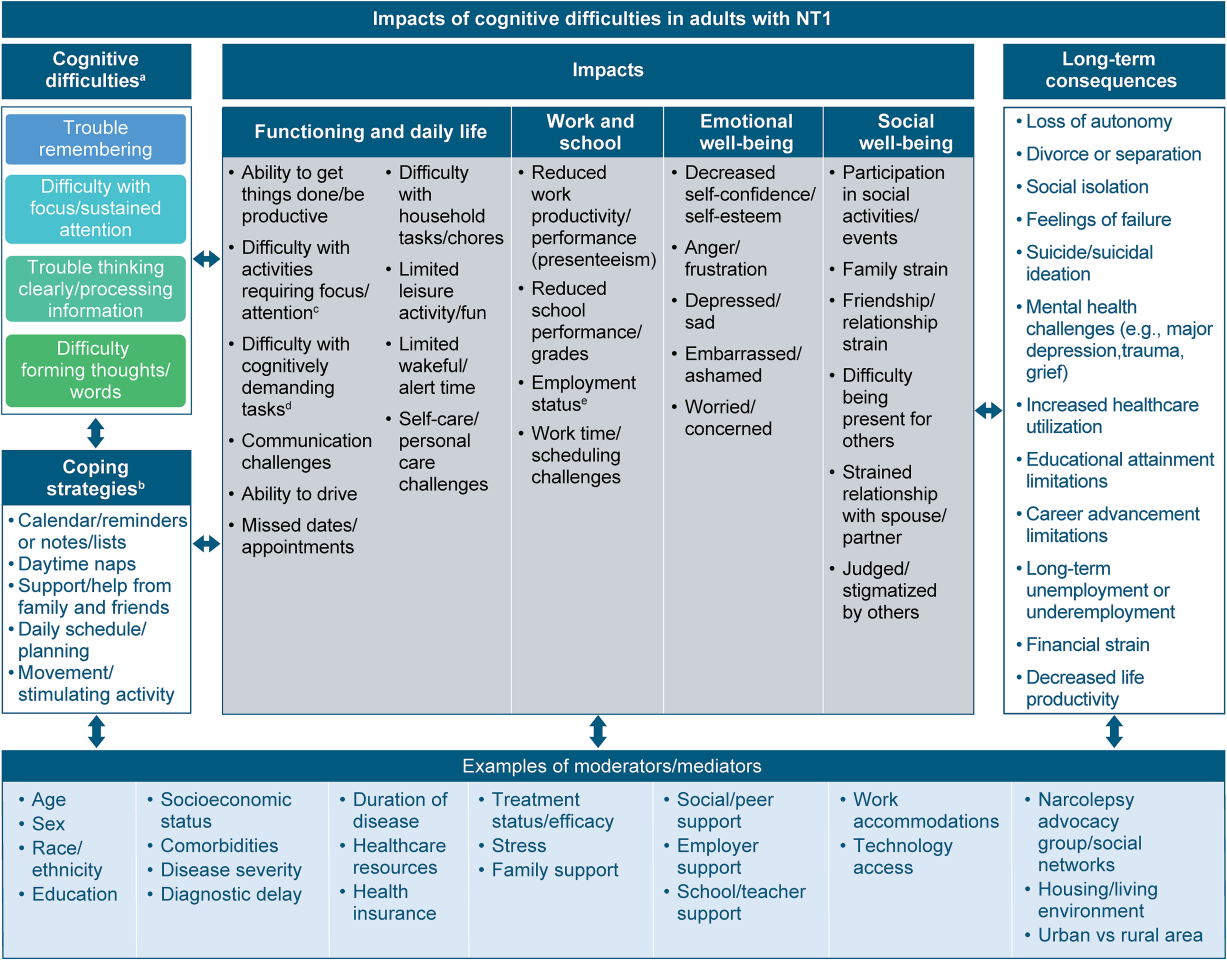
Theoretical model of the impacts of cognitive difficulties in adults with NT1. ^a^Includes the most frequently reported cognitive symptoms (>50%) among participants. Difficulty learning was reported by 13 (28%) participants; “Brain fog” was a more general concept that participants used to refer to various cognitive difficulties. ^b^Includes cognitive difficulty management/coping strategies most often mentioned by participants. ^c^For example, reading, writing, watching TV/movies, listening to audiobooks/podcasts. ^d^For example, planning, making decisions, complex reasoning. ^e^For example, job loss, inability to work. NT1, narcolepsy type 1.

Based on the outcomes from the qualitative analysis, a conceptual model of the experiences of cognitive difficulties, their broad impacts on daily functioning and well-being, and their long-term consequences in adults with NT1 was developed (Figure 4). Impacts in each domain were considered major if they were reported by at least 20% of participants for at least 1 of the key cognitive difficulties in the model and were considered minor if they were reported by fewer than 20% of participants for all key cognitive difficulties, but by at least 10% of participants for at least 1 key difficulty. Examples of possible moderating or mediating factors were also depicted. Examples of moderators (ie, factors that may increase or lessen the relationship between a cognitive difficulty and its impact or consequence) include age, socioeconomic status, and severity of NT1, while examples of mediators (ie, factors that may explain the relationship between cognitive difficulties and impacts or consequences) include stress, family support, and employer support.

The theoretical model was refined based on input from the focus group. Focus group participants confirmed the relevance, accuracy, and comprehensiveness of the conceptual model, including the symptoms, impacts/consequences, and coping strategies identified. Participants particularly appreciated the inclusion of concepts that have previously received limited attention in the medical literature on NT1, including cognitive difficulties. As one participant explained:

*“Looking at the symptoms…it's so good to see cognitive functioning included within that because I feel like we get that. The brain fog, the cognitive issues get lost in the conversation, because we're so focused on medical literature…focusing on the pentad [of narcolepsy symptoms], but it's more than just that. It's more than cognitive functioning too, but I'm glad to see that this is receiving a lot of attention in the work that you're doing.”* (male, age 41 years)

Minor edits to some of the language in the model were made based on focus group participants’ suggestions to avoid language that could potentially be considered offensive. For example, they suggested replacing the long-term consequence of “mental health issues” with “mental health challenges,” as the former term was identified as potentially stigmatizing.

## 4 Discussion

This secondary analysis used data from a large, in-depth qualitative interview study of adults with NT1, which examined the broader lived experience of the condition; the present analysis focused specifically on cognitive difficulties and their consequences for daily life and overall well-being. Although existing research in adults with NT1 has consistently demonstrated the prevalence and burden of cognitive symptoms, important gaps remain in understanding how these symptoms are described and experienced in daily life. These gaps included limited understanding of (1) the relationships between cognitive difficulties and their impacts on daily life, (2) the frequency and perceived severity of cognitive difficulties, and (3) specific cognitive difficulties underlying the commonly used but ill-defined term “brain fog.” The current study extends previous research (5–8, 18) by providing deeper understanding of the cognitive difficulties reported by adults with NT1 and their impacts on everyday life, based on rich qualitative data from the patient perspective. In-depth qualitative interviews enabled insight into aspects of the cognitive experience and consequences that are not readily captured with surveys or qualitative methods. The findings also provide important context for emerging neuropsychological models of NT1.

The findings from this study are consistent with previous research that shows cognitive symptoms to be one of the most commonly reported and disruptive symptoms of NT1 (5, 19, 20). The key cognitive difficulties described by the adults with NT1 interviewed here included difficulty with focus/sustained attention, trouble thinking clearly/processing information, difficulty forming thoughts/words, and difficulty learning new things, with a high rate of co-occurrence of descriptors used by participants. These specific concerns are consistent with subjective reports collected in other NT1 studies. In Maski et al., 46.9% of NT1 survey participants responded that difficulty thinking, remembering, concentrating, or paying attention were among the most significant symptoms that impacted life (19–21). Likewise, a high number of NT1 participants using Narcolepsy Monitor, a mobile app to rate burden of symptoms reported such cognitive difficulties: 93% reported concentration difficulties and 91.4% reported memory difficulties (22, 23). Our findings also align with objective cognitive data collected from people with NT1 reported in the literature. A recent meta-analysis of cognitive outcomes reported large impairments in attention [Cohen’s d (*d*) = −0.9] and small to moderate impairments in executive function (*d* = −0.3) and learning and memory (*d* = −0.33) (7). Given the consistency observed in the data, our findings support a shift toward more specific and clinically meaningful language to describe cognitive difficulties, rather than reliance on the nonspecific term “brain fog”.

Participants described how cognitive difficulties interfered with everyday functioning, including at school and work. These accounts provide context for understanding how cognitive symptoms may be relevant to economic and productivity outcomes. For example, European studies report lower employment among people with narcolepsy (22), and other research has documented significantly higher levels of work productivity loss, including presenteeism, absenteeism, and overall activity impairment relative to peers not diagnosed with narcolepsy (23). Previous studies have also identified associations between workplace absenteeism and presenteeism and symptoms such as excessive daytime sleepiness, depression, and anxiety in narcolepsy (24). While the present findings do not establish causal relationships, the conceptual model developed in this study suggests that cognitive symptoms likely contribute to academic and occupational challenges. This study also highlights coping strategies, including daytime naps, seeking stimulating activities, and support from family and friends, which participants reported as helping to manage daily demands and improve productivity.

Participants also described ways in which cognitive symptoms impact social and family relationships, as well as self-confidence and mood. For example, one participant described how difficulty recalling important information shared by her child contributed to feelings of guilt and strain within the family. Other participants reported withdrawing from social interactions due to difficulty sustaining attention during conversations, leading to frustration and reduced social engagement. Existing literature in narcolepsy has documented associations between depression and anxiety and overall disease severity measured by the Narcolepsy Severity Scale, as well as subjective excessive daytime sleepiness (25, 26). While these factors are known to play an important role in social and emotional well-being, the qualitative data presented here suggest that cognitive symptoms are also relevant.

### 4.1 Strengths and limitations

A strength of this study is that it was based on a large sample of adults with NT1. The involvement of a researcher living with NT1 (SLB) in all stages of the research process, including conceptualization, study design, data interpretation, and writing, allowed for enhanced understanding and interpretation of participant experiences. Additionally, use of patient-created content in the development of the discussion guide ensured that a broad range of experiences important to people with NT1 could be captured. The in-depth 90-min interviews allowed for deep exploration of participant experiences of NT1-associated cognitive symptoms.

This study also had some limitations that should be considered. The sample was predominantly female and living in the United States, potentially limiting the generalizability of the study findings to other populations. The nature of the study introduces the potential for participant selection bias, whereby those who agreed to participate may differ from those who did not. Participant responses may be subject to recall bias, particularly given that participants sometimes discussed impacts that occurred years prior to their interview. Additionally, treatment status and treatment types varied widely among the participants, which may have affected the study findings.

## 5 Conclusion

To our knowledge, this analysis is the most comprehensive, in-depth qualitative investigation of patient-reported cognitive difficulties in adults with NT1 to date. In this sample, cognitive difficulties, most commonly described as trouble remembering and difficulty with focus/sustained attention, were frequent and disruptive, and were associated with wide-ranging and consequential impacts on adults’ lives. The development of a conceptual model based on participant experiences from this research has the potential to improve clinicians’ understanding of the language used by adults with NT1 to describe the experiences of their cognitive symptoms and the associated impacts, which can be used to inform clinical practice and future research, as well as to offer a model for aligning cognitive difficulties with standardized assessments of cognitive impairment. The findings from this patient-centered study of cognitive difficulties in adults with NT1 accord with the cognitive impairments observed in neuropsychological studies. However, there is still a need to better understand how the described experiences of cognitive difficulties reported here align with evidence based on standardized assessments of cognitive impairment to better understand cognitive symptoms associated with NT1.

## Data Availability

The datasets presented in this article are not readily available because this would exceed the scope of participant consent and privacy agreements. Representative quotations have been disclosed in the manuscript. Additional datasets generated during and/or analyzed during the current study are available from the corresponding author on reasonable request. Data will be shared only after deidentification, in compliance with applicable privacy laws, data protection regulation, and consent requirements. Requests to access the datasets should be directed to GlobalPublications.Data@takeda.com.
