# Supplementary material for: Patient-reported experiences of cognitive difficulties and their impact on daily life in narcolepsy type 1
Source: Front Neurol. 2026 Mar 4;17:1760952. doi: 10.3389/fneur.2026.1760952 (PMC12996208; doi:10.3389/fneur.2026.1760952)
Supplement: Supplementary file 1 [file Supplementary_file_1.docx]

**Supplementary Information**

**Supplementary Table 1**. Cognitive difficulties in NT1 (additional selected quotations)

| **Symptom themes** | **Selected quotations** |
| --- | --- |
| Trouble remembering | *“…I used to pride myself…on being, you know, very intelligent…and now I, I struggle with…simply remembering…things like, uh, what we did the day before for, for dinner, or…me and my wife may have, have talked about something that we planned to do…on the weekend and I don't recall exactly what we talked about.”(*male, age 35 years)  “Forgetfulness has always been something…I've always struggled with, like, since I can remember.” (female, age 42 years)  “…the sleepiness can be overwhelming. Um, you know, I'll forget. I can be in the middle of something and forget what I was doing…I'll think, you know, "Hey, I need to do this later," but by the time later runs around I may forget.” (female, age 32 years) |
| Difficulty with focus/sustained attention | *“…it's difficult to focus.”* (female, age 42 years).  “…when I'm tired, if I'm learning something or paying attention to something, I'm not really able to pay attention or comprehend the things that they're saying.” (female, age 29 years)  “There's times where like people are talking to me. And I just like zone out. And I know it's happening. But I can't kind of get with it, can't get my brain to like fire on all cylinders.” (female, age 27 years) |
| Trouble thinking clearly/processing information | *“…my brain is, like, gray. There's just not a lot going on, and it's kind of like everything is . . . It's like a fog in my mind, in a way…it takes a lot to get to a conclusion or to a thought...”* (female, age 24 years).  “…I feel like I'm just not as creative…as I used to be. I'm not as…spot-on as I used to be. I'm not as sharp.” (female, age 42 years)  “…a sleep attack is when I get so tired and my brain immediately shuts down that I can't process. And the only thing it can think about is sleep.” (female, age 37 years) |
| Difficulty forming thoughts/words | “…you're not able to form the thoughts and form the feelings that you are experiencing in a healthy way because maybe you need longer than most people.” (female, age 27 years)  “Every once in a while, like I won't be able to think of the right wor -- a word.” (female, age 54 years)  **“I forget words all the time now, too, and that's horrible because I love my words.” (female, age 39 years)** |
| Difficulty learning new things | “I do have issues kind of picking up on things the first time. It takes me a little bit.” (female, age 27 years)  “Just when I'm tired, if I'm learning something or paying attention to something, I'm not really able to pay attention or comprehend the things that they're saying.” (female, age 29 years) |
| Brain fog | “[Brain fog is] Um, like kind of hazy-feeling. Like…I can't focus, can't grab a thought, can't stay focused on anything. Or I'll lose words like that. You know? That's what I mean.” (female, age 56 years) |

NT1 = narcolepsy type 1.

**Supplementary Table 2**. Impacts associated with cognitive difficulties in NT1 (additional selected quotations)

| **Symptom themes** | **Selected quotations** |
| --- | --- |
| Impacts on functioning and daily life | *“It can be difficult especially in conversation with people. And I don't like -- even at my most severe sleepiness, I've never like fallen asleep talking to someone. But just paying attention and being invested in a conversation can be very difficult.”* (female, age 20 years).  *“I’m a huge reader. I love to read. I used to be the person who would read three different books at the same time because they were three different genres. And now, there are some books that I can’t read anymore because the [number] of times I have to go back to reread…because I forgot what I just read…That’s horrendous.”* (female, age 40 years, untreated).  *“…when you can't think it's hard to function. Yeah, mainly that. Like, when you just can't think straight…it's just really hard to get things done.”* (female, age 28 years).  *“[I have] Difficulty concentrating…sometimes difficulty reasoning through things…especially if there are like multiple processes…feeling overwhelmed.”* (female, age 42 years). |
| Impacts on work and education | *“I'm forgetful. That's a big one…like, I'll forget things like -- people [at work] will say, "Well, you do this, this, and this," …and tell me in great detail, and I can't remember any of it.”* (female, age 56 years).  *“…if I have to…focus or have sustained attention for a long period of time, then I kind of…get really tired. And I c-catch myself… zoning out or-or not paying as much attention as I should. Like it's hard for me to sit through meetings…”* (female, age 54 years).  *“[Brain fog is] probably the worst in the morning…but I definitely do experience it…in the afternoon as well…it can be very difficult especially…with my job, if I'm seeing patients… or… working with my team and trying to… work on 5,000 things at once…it can be very difficult to try and get the work done that I want to… and to try and perform at the level that I want to for my job.”* (female, age 35 years).  *“…in undergrad, I really struggled in classes. Like, my memory -- I couldn't memorize stuff. I couldn't sit for long periods of time to study, yeah, all of that. I just -- I went to class every day and I would fall asleep within the first five minutes. So I wasn't really retaining anything and didn't really learn much.”* (female, age 29 years).  *“…when I do experience [a loss of concentration] …it's, uh, typically…if I'm doing a long reading for a class or if I'm, uh, taking an exam or something, then I'll start to -- it's more so that I'm like -- I'll be reading the content but not actually comprehending it or absorbing any of it. So…I'll realize halfway through and then have to go back and start over again.”* (male, age 22 years). |
| Impacts on emotional well-being | *“I think [having narcolepsy] lowers my confidence at times at work because of the whole, like, difficulty speaking and thinking.”* (female, age 30 years).  *“Sometimes it's really annoying to not be able to listen. So like, if someone is trying to tell me something or -- like in a group story…I get zoned out…So that can be frustrating because…I want to be in tune and I want to be in touch, but it's kind of hard to do so sometimes.”* (female, age 20 years).  *[Interviewer: “What is embarrassing about narcolepsy?”] “ Um, not being present when I'm supposed to be. Kind of like-like I don't know, it just really…feels like I've let myself down every time [it] happens and that I'm like being lazy…”* (male, age 37 years).  *“…when I have the cycles of insomnia, it is, like, almost impossible to function during the day. And so I either spend the whole day trying to sleep and failing, um, or I try to do what I would normally do in a day, and I just can't have normal conversations with people. I can't get anything done. I don't have focus. I don't remember anything. Um, and then it affects, like, my mental health a lot when I have insomnia -- I get pretty sad and just, like, really confused.”* (female, age 24 years). |
| Impacts on social well-being and relationships | *“Well, there's a lot of times, you know, my wife asks me to do things, and I tend to forget quite a bit. And it's not really out of fault of me so much as, you know, I really do forget.”* (male, age 27 years).  *“…there's been things even that like my son has told me -- like, he'll tell me something -- like, he'll mention offhand something about school, and I'll say, "Oh my gosh, that's so cool. Like, why didn't you tell me that before?" And he says, "Mom, I did." You know? And it's the kind of thing that like is really meaningful for me, and for him, and for us as a family. And so, I'm like -- I really should've remembered that one.”* (female, age 46 years).  *“…[Brain fog] will happen often…as we approach the 9:00 hour, um, at night, or 9:30 PM if I'm -- if I've had a very busy day and, like, we have company and they keep talking to me, and I'm trying to . . . I don't know if my brain just turns off as it knows it's time for bed. Um, but it's like I can no longer think of anything to say to the person or reflect what they're saying or, um, share a thought. It's like there's just air in my brain.”* (female, age 30 years). |

NT1 = narcolepsy type 1.
